# Evaluating the effectiveness of participatory science dog teams to detect devitalized Spotted Lanternfly (*Lycorma delicatula*) egg masses

**DOI:** 10.7717/peerj.19656

**Published:** 2025-07-16

**Authors:** Sally Dickinson, Mizuho Nita, Edgar O. Aviles-Rosa, Nathan Hall, Erica N. Feuerbacher

**Affiliations:** 1 School of Animal Sciences, Virginia Polytechnic Institute and State University (Virginia Tech), Blacksburg, VA, United States of America; 2 School of Plant and Environmental Sciences, Virginia Polytechnic Institute and State University (Virginia Tech), Winchester, VA, United States of America; 3 Animal and Food Science Department, Texas Tech University, Lubbock, TX, United States of America

**Keywords:** Spotted lanternfly, Conservation detection dogs, Detection dogs evaluations, Participatory science, Community science, Invasive species detection

## Abstract

The spotted lanternfly (*Lycorma delicatula*, SLF) is an invasive planthopper first detected in the United States in 2014, with initial sightings in Pennsylvania. SLF poses a serious threat to agriculture, particularly targeting grapevines, hops, and ornamental plants, resulting in substantial annual economic losses. Due to its life cycle, the early detection and removal of egg masses are the most effective strategies for preventing long-distance dispersal. However, visual detection by humans is time-consuming and inefficient. Detection dogs have demonstrated high accuracy in locating SLF egg masses and differentiating them from environmental distractors. Despite their effectiveness, the number of dogs available through governmental channels is insufficient to meet demand. This study evaluated whether community scientist dog-handler teams could meet standardized detection criteria using SLF egg masses. Teams from across the U.S. were recruited and trained using devitalized egg masses, with oversight provided by local trainers. Following a 3- to 6-month independent training period, team performance was assessed through an odor recognition test and a field trial. Dogs demonstrated a sensitivity of 82% in controlled testing and 58% in field conditions. These results provide proof of concept; community scientist dog teams could play a significant role in protecting their local environments and agriculture from invasive species.

## Introduction

Dogs have been successfully trained to detect a wide range of target odors, including cancer cells in human tissue samples (Guerrero-Flores et al., 2017), water treatment chemicals in soil to isolate water main leaks (Zhong & Chen, 2023), body-worn explosives in the aerodynamic wakes of moving individuals (Lazarowski et al., 2018), and specific viruses for rapid patient screening (Otto et al., 2023). A growing subset of detection dogs, known as conservation detection dogs (CDDs), operates in conservation contexts, performing tasks to identify ecologically relevant target odors (Grimm-Seyfarth, Harms & Berger, 2021). These targets may include living organisms or their remnants. For instance, CDDs have been deployed to detect the Great Crested Newt (*Triturus cristatus*) underground in their natural habitats (Glover et al., 2023). They have also been trained to locate nests, residues, and scat. For example, dogs detected the scat of black bears (*Ursus americanus*), fishers (*Pekania pennanti*), and bobcats (*Lynx rufus*), enabling researchers to estimate the population densities of forest carnivores (Long et al., 2007). Furthermore, CDDs have been used to detect scat from sentinel species, such as mink and otter, providing ecologists with data on health, genetics, and environmental contaminants, including heavy metals and pharmaceuticals (Richards et al., 2018). In addition to conservation tasks, CDDs have proven valuable in agricultural contexts. One such application is the detection of the Spotted Lanternfly (*Lycorma delicatula*, SLF), an invasive planthopper native to China, India, and Vietnam. Spotted Lanternfly were inadvertently introduced to Pennsylvania, USA, likely through a shipment of landscaping stone containing SLF egg masses in 2014 (Urban, 2020). Its population rapidly expanded due to the absence of natural predators, the abundance of suitable host plants (Dara, Barringer & Arthurs, 2015; Urban, 2020), and human activities that facilitated its spread (Essler et al., 2021; Bullock, Moylett & Para, 2017).

The Spotted Lanternfly has six life stages: egg masses, four nymph stages, and the adult stage. For two reasons, the egg mass stage is the ideal target for detection efforts. First, SLF adults often attach egg masses to wood and stones, which serve as a significant vector for SLF relocation, enabling the species to invade new habitats. Second, each egg mass contains 30 to 60 eggs (Dara, Barringer & Arthurs, 2015), making mass eradication more feasible than targeting individual nymphs or adults. While humans can be trained to locate SLF egg masses, many of these masses are concealed in small crevices, high in tree openings, on the underside of lumber, or in other inconspicuous locations. As a result, human detection is often impractical without an extensive time investment. In contrast, CDDs can accurately detect egg masses on concealed surfaces without disturbing or damaging the material during inspection. Several studies have demonstrated the feasibility of using dogs for detecting SLF egg masses, highlighting their efficiency in locating hidden infestations (Keller & Hoover, 2023). Aviles-Rosa et al. (2023) used shelter dogs to evaluate their ability to discriminate devitalized egg masses from other ecologically relevant distractor odors. Similarly, Essler et al. (2021) demonstrated that three trained search-and-rescue dogs could successfully transition to detecting SLF egg masses, achieving a sensitivity of up to 94.6% and a specificity of 92.8%. Extending these findings to real-world applications, Fuller et al. (2024) compared canine-assisted surveillance to human searches for early SLF detection and found that trained detection dogs significantly outperformed humans in transect searches, locating more egg masses with greater efficiency. Together, these studies highlight the reliability and practical advantages of using trained dogs for SLF egg mass detection across both controlled and field environments.

Despite the documented utility of conservation detection dogs (CDDs), Rutter et al. (2022) highlighted the significant financial burden on localities and agencies for procuring, training, maintaining, and deploying professional CDDs. Often, agencies can afford only one or two dogs, forcing them to prioritize target odors for training. During the early stages of the SLF infestation, there were far fewer conservation detection dogs (CDDs) than needed. CDDs from various groups, including the Pennsylvania Department of Agriculture, were trained to detect SLF egg masses and worked effectively in the field. However, the continued expansion of the SLF population highlighted the need for greater availability of trained detection dogs. One potential strategy for addressing the shortage of CDD teams is to leverage companion dogs and their non-professional handlers to expand capacity. Research and sport scent work communities have demonstrated that dogs from a wide range of breeds, including those not traditionally used in detection work, can perform successfully in scent detection tasks. For example, in sport scent detection trials, dogs of all breeds and sizes have excelled (NACSW, 2024b). Supporting this further, Hall et al. (2015) found that pugs, a brachycephalic breed, outperformed German shepherds in a controlled laboratory detection task, highlighting that detection aptitude is not limited to traditionally favored breeds. Similarly, Rutter et al. (2021) trained volunteers and their companion dogs of various breeds and training backgrounds to detect a novel odor (myrrh essential oil) in just 12 weeks, achieving a minimum of 75% correct responses. The growing popularity of sport scent detection through networks like the National Association of Canine Scent Work (NACSW, 2024a) who, in 2018 reported a membership of 19,527, with 241 different breeds represented (NACSW, 2018) suggests that a subset of this community could be mobilized for conservation efforts. Many participants may welcome the opportunity to apply their skills to meaningful volunteer activities that protect local environments. These findings highlight the potential for non-traditional, non-purpose-bred detection dogs to play a significant role in conservation contexts.

The purpose of this proof-of-concept study was to evaluate whether a participatory science model, specifically, community scientists and their companion dogs with prior experience in sport scent detection competition, could be trained to detect devitalized SLF egg masses with accuracy comparable to that of professional detection dog teams (Association for Advancing Participatory Sciences, 2024). Additionally, we examined whether these teams could generalize from devitalized to live SLF egg masses. Dogs were first assessed using an odor recognition test (ORT) and a field evaluation (FE), both of which were modeled after procedures used by professional detection teams, to validate their initial training. Teams that successfully passed both the ORT and FE were subsequently evaluated on a second ORT designed to assess their ability to detect live SLF eggs, following minimal additional training.

## Study Overview

**Figure 1:**
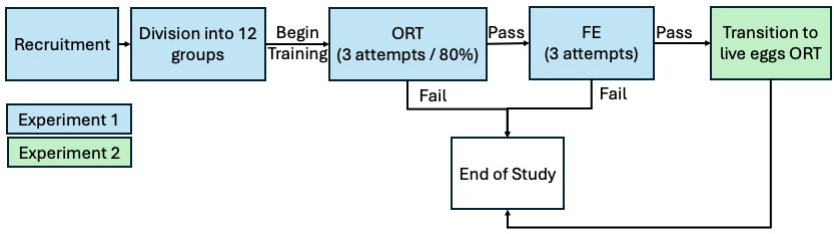
Participatory science dog team SLF detection study overview. Participants were given three attempts to achieve an 80% passing score on the ORT, FE, and LE-ORT. Full details of the evaluations are listed in Experiments 1 and 2.

This study consists of two sequential experiments to evaluate the effectiveness of participatory science dog teams in detecting SLF egg masses. Experiment 1 assessed the ability of recruited teams to detect devitalized SLF egg masses in an Odor Recognition Test (ORT) and a Field Evaluation (FE) after they had completed training on devitalized egg masses. A subset of teams that successfully completed the ORT and FE proceeded to Experiment 2, which assessed their ability to transition from detecting devitalized SLF eggs to live SLF eggs, within the quarantine zone only. The progression and interrelationships of these experiments are illustrated in Fig. 1.

## Experiment 1. Odor Recognition Test and Field Evaluation

### Methods and Materials

#### Participants

Approval to conduct this study was granted by the Virginia Tech Institutional Animal Care and Use Committee (IACUC #23-025) and the Virginia Tech Institutional Review Board (IRB #23-257). Through Facebook posts, we recruited potential dog-handler teams with experience in either sport scent detection competition or other detection work (*e.g.*, search and rescue, bed bug detection). We invited the handlers to attend an informational webinar, which they could attend live or watch *via* recording. After watching the webinar, those interested in participating completed a Google form in which they self-reported their own and their dog’s scent detection experience, whether they wanted to be considered as a handler and/or a trainer, and their geographic location. From the participants who completed the Google form, we created groups of handlers (owners who would train and test their dogs) with an overseeing trainer based on geographic proximity. All participants signed a written consent form, agreeing to participate in the study. The trainers served as the primary point of contact between the research team and the dog-handler teams, receiving and distributing materials, organizing training sessions, and coordinating evaluations. Many of the handlers and trainers had a prior teacher/student relationship. In all teams, the trainer also participated as a handler for at least one dog in the study. We enrolled all participants in a Google Classroom space, where they could access the forms, questionnaires, and study updates. We also invited all participants to join an optional private Facebook group, which facilitated a sense of community and allowed teams to ask questions about any aspects of the study.

#### Questionnaires and training log

Each handler was asked to complete five online questionnaires. Links to the questionnaires were provided to handlers in the Google Classroom; handlers were encouraged to complete them at the time of enrollment; however, many handlers completed them at various times throughout the study. Handlers were also encouraged to record their training weekly *via* a Google form. The form included the handler’s and dog’s names for identification, the number of training sessions per week, the average length of each training session, and the intended objective of the training. The results of the surveys and training logs will be analyzed in a separate paper, which will discuss the predictive factors for teams’ success in this study.

#### Training aids

**Figure 2:**
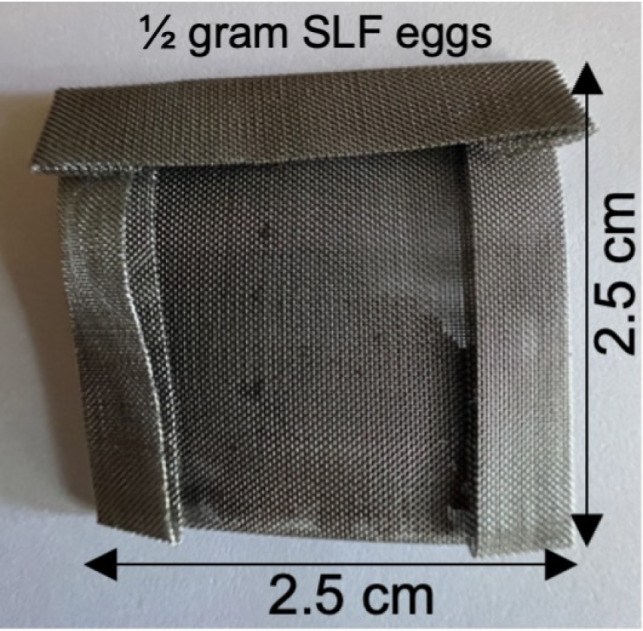
Picture of devitalized SLF egg training aid (SLF-training aid). Constructed of folded and crimped stainless steel mesh with 0.5 g devitalized SLF eggs.

We harvested approximately 1,500 grams of SLF egg masses over two consecutive winters at the Virginia Tech Agricultural Research and Extension Center in Winchester, Virginia. The eggs were scraped from the substrate using a plastic scraper and directly transferred into glass jars, each holding 30 to 50 grams of eggs. The sealed glass jars were stored in a −80 °C freezer for at least 30 days to ensure the eggs were devitalized, rendering them inert and suitable for shipping outside the quarantine zone (Essler et al., 2021). We cut, folded, and crimped 304-grade stainless steel 80-mesh into 2.5 cm × 2.5 cm packets to create the training aid packets for distribution to the teams. The training aid packets were designed to provide a consistent odor sample for the teams to train with while ensuring no SLF eggs were dispersed into the environment (Kane, Aviles-Rosa & Hall, 2023). We washed the empty packets in methanol to remove manufacturing oils and other contaminants. Then, we baked them in a 200° Celsius oven to evaporate the remaining methanol and allowed the packets to air cool. We added 0.5 g of devitalized eggs to each packet and then folded and crimped the top flap to secure the eggs inside, creating the Spotted Lanternfly training aid (SLF training aid), as shown in Fig. 2. The SLF training aids were placed in sealed Mylar bags and stored in a residential freezer at −20 °C until shipped to the teams. We also isolated a subset of the cleaned packets without any enclosed eggs to serve as matched blanks (MB) for training and testing, ensuring that dogs were alerting to SLF egg-specific odor stimuli. We placed the MB in sealed Mylar bags and stored them separately at room temperature to ensure zero odor transfer. We shipped training packs overnight to the trainers. Each pack contained six SLF training aids and one MB per handler. We also included instructions on storing the SLF training aids in sealed Mylar bags in a freezer when not in use, as well as guidelines on recording the number of hours the SLF training aids were out of the freezer for training purposes. Handlers were instructed to use a new SLF training aid after 40 h of use. Additional shipments of SLF training aids were made as needed.

#### Training

We provided no instructions or requirements on how the training should be conducted, allowing the trainers to work with their groups as usual. The handler completed a weekly training log for each dog participating, which was submitted *via* a Google form. Handlers reported the number of training sessions they finished, the average length of each training session, the training method used, and the focus of the training sessions. Trainers communicated to the research team when their teams were ready to begin evaluations, and then the research team coordinated with them to establish a testing time and location.

#### Odor recognition trial

We conducted the odor recognition test (ORT) at locations identified by the trainers. In some instances, these locations were familiar to the dogs; in others, they were novel.

**Figure 3:**
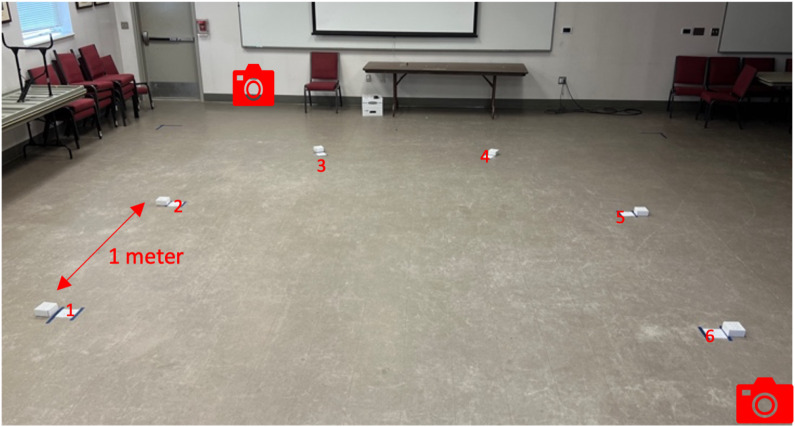
SLF ORT Set up. Picture showing the typical ORT setup with camera locations, box spacing, and numbers in the array.

**Setting.** All ORTs were conducted in a space with a minimum of 7 m × 7 m of unobstructed open area, inside or outside. The Evaluator assessed the space for safety and the absence of significant odors such as air fresheners or other distractions such as accessible food. The Evaluator created the trial array by placing numbered cards (1–6) on the floor and securing them with tape, 1 m apart, in a semicircle configuration (Fig. 3). These cards designated where the boxes would be placed during the trials. We placed two GoPro cameras, a minimum of two m away from the array, on the outer edge, proximal to positions three and six, to record all trials.

**Evaluator and observers.** For each ORT, a specific person was designated the Evaluator, responsible for administering the ORT. In some cases, the Evaluator was one of the researchers, and in other cases, this role was filled by the trainer or a designee if the trainer was handling the dog. Additional observers were present for all ORTs; only the Evaluator could access the testing sheets. All observers remained quiet and behind the Evaluator during the trials. During ORT trials, no other dogs were allowed in the space. The Evaluator was positioned at the front of the room and, during the trial (from the beginning of the search until the handler notified the Evaluator of their findings), maintained their back toward the team conducting the test. This procedure ensured the trials were consistently double-masked. The Evaluator was responsible for reviewing the protocol with each testing team, placing the boxes in the correct position during each trial, and recording the time and outcomes of each trial on the test sheet.

**Boxes and odors.** During the setup time on the ORT day, the Evaluator prepared two sets (A and B) of seven cardboard boxes each; each box measured 10 cm × 10 cm × 5 cm. We drilled nine 6.35-mm holes in the top flap, arranged in a 3 × 3 pattern, and labeled each box with a unique code on the underside to designate its contents. One box contained the SLF training aid, and the other six boxes contained distractor odors as detailed: one contained the MB, one contained locally sourced tree bark, one contained locally sourced grass, one contained a medical glove, and two remained empty. Between trials, the boxes were placed in designated spaces on a tray with the holes oriented down. To further isolate the SLF egg odor, the SLF training aid box was placed with holes down, and at a minimum of 15 cm space between it and the other boxes. We prepared three additional training boxes for the teams to use during the acclimation period before the first trial commenced. One acclimation box contained the SLF training aid, one contained the MB, and one was empty. The boxes were clearly labeled on top, so the handlers could easily identify the correct one. Additionally, empty boxes were available to exchange during the trials if a box became damaged or contaminated. The Evaluator decided when to exchange a box.

**ORT setup.** Each ORT began with the Evaluator orienting the handler, without the dog present, to the room, the numbered spaces on the floor, and how the handler should signal an alert by the dog to the Evaluator. The Evaluator also reviewed the protocol, including the number of boxes per trial, the number of target odors, the number of distracting odors, and time limits, with the handler. Afterward, the handler was allowed to ask questions. When ready, the handler returned to the ORT area with their dog on a leash and was given a 2-minute acclimation period. During this time, the three marked training boxes, one with an SLF training aid, one with MB, and one empty, were placed outside of the array area (no other boxes were in place at this time). The handler was instructed that they could use the acclimation time however they wanted. For example, they could cue and reinforce the correct choice, perform a practice search of the three boxes, or use the time to allow the dog to become comfortable in the space by engaging in other activities that provide reinforcement. After the acclimation period, the handler and dog moved to the inter-trial team space, a minimum of 5 m away from the array, and had water available for the dog throughout the test. The handler and their dog stood in the inter-trial team space, their backs to the array. Once the handler was facing away, the Evaluator removed the acclimation boxes, placed them with the hole-side down on a tray outside the array, and prepared for the test.

**ORT test.** Each ORT test was made up of 10 trials. Each trial contained zero or one SLF training aid and five or six distractor odors. For each trial, the placement of the specific odors was predetermined, and we created four different 10-trial test versions. The four test versions were constructed using a computer randomizing program. Before the acclimation time, the handler drew a card to determine which version they would run; however, they were unaware of the version number. If the handler was running multiple dogs, the version they ran the previous time was removed from the options for their subsequent dog. The Evaluator placed the boxes from set A at the designated positions on the floor. Boxes were always placed in positions one through six sequentially and then picked up in the same order to ensure that no specific odor was ever handled last or first. Once the trial boxes were in place, the team was instructed to begin the trial. The open end of the horseshoe array was considered the start line, located between boxes one and six. The handler and dog could choose which direction to work in and whether they would be on or off-leash. The handlers could carry the dog’s reinforcer (usually treats or a toy) with them and were permitted to provide it within the array, however, and whenever they chose. Teams had 90 s per trial, which started when the handler cued the dog to search. The trial ended when the handler called out “alert”, or the box number for an odor find, “clear” if they determined no target odor was present, or when 90 s had elapsed. The handler was responsible for determining if the dog showed an alert. After the trial, the evaluator provided the correct outcome, regardless of whether the team succeeded. The team then returned to the inter-trial space, and the Evaluator reset the array for the subsequent trial until 10 trials were complete or the handler opted to withdraw for any reason. Between trials five and six, box set A was replaced with box set B to ensure neither the dog nor the handler was learning the specific odor or look of the SLF training aid box in set A. After a team completed their ORT, all boxes were inspected and replaced if they were deemed contaminated or visually identifiable. The floor was checked for food or other debris and cleaned as appropriate. The time between testing teams was at least 10 min to allow for reset and preparation for the next team.

**Measurement.** The Evaluator recorded the handler’s call for each trial, indicating whether it was correct, along with the time elapsed from the start of the trial to when the handler called out “find” or “clear”, or if 90 s elapsed with no determination made by the handler (no response). Video recorded on the GoPro camera was available to verify times. To pass the ORT, the team must correctly complete at least eight out of ten trials. A team that failed to meet the 80 percent requirement was given two additional opportunities to retake the test, either on the same date or at a future date. If the team retested, the version(s) they had run previously were removed from the options.

#### Field evaluation

Teams that passed the ORT could attempt the field evaluation (FE). As with the ORT, the FE was conducted at locations identified by the trainers. In some instances, the dogs were familiar with these locations; in others, they were novel.

**Figure 4:**
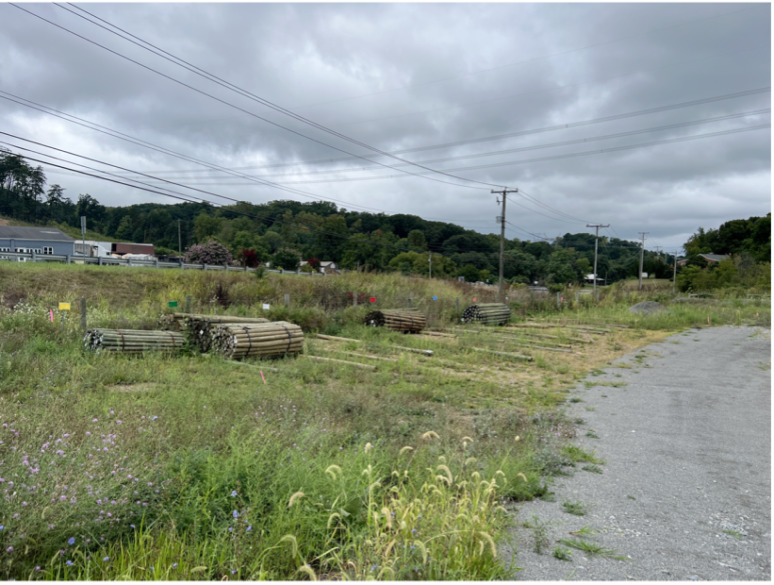
Typical of FE area. The photograph shows a typical FE search area, including lumber and other objects.

**Setting.** The FE was conducted in an exterior or semi-exterior space (open barn, picnic area, lumber yard, *etc*.) (Fig. 4) that was a minimum 25 m × 25 m area, with safe access to the entire area. The Evaluator assessed the space for safety and evidence of SLF egg masses. We placed cones or flagging tape to designate the corners of the search area. We recorded using two GoPro cameras; one was positioned to have visibility over the evaluation area, and the other was worn on a chest harness by the handler.

**Evaluator and observers.** The Evaluator functioned just as they did for the ORT. In some cases, the Evaluator was one of the researchers; in other cases, this role was filled by the trainer or their designee. On all FE trials, additional observers were present; however, only the Evaluator knew the hide locations. Observers were positioned a minimum of 10 m outside the search area and remained quiet during the trial. No other dogs were allowed in or around the search area during FE trials. The Evaluator remained outside the search area but close enough to communicate with the handler. The Evaluator had their back turned while the team searched to ensure the trial was double-masked. The Evaluator was responsible for reviewing the protocol with each testing team, placing the SLF-training aids and MB in the search area, and recording times and outcomes of the FE on the test sheet.

**FE odor setup.** We placed the SLF-training aid and MB in a 44 mL glass jar with no lid to prevent odor transfer to the surrounding substrates. We could reuse an area for a different dog team by doing so. Between three and five SLF-training aids were placed in the search area; however, the team and all observers were masked to the number and placement of the hides. The Evaluator determined SLF-training aid and MB placement in the search area, considering weather conditions and the availability of suitable concealment locations, with care taken to prevent overlapping odors from adjacent hides. One hide was placed at ground level, one at a maximum height of 1 m, and the others at heights equal to or between these extremes. All hide locations were placed so the handler and the dog did not have to climb on, over, or crawl under anything. The SLF-training aids were concealed in the environment out of the handler’s plain sight. The Evaluator created a sketch of the search area, numbering the SLF-training aid hide and MB locations, which correlated with the hide numbers on the test sheet and allowed for easy recording of times during the search. If the handler ran multiple dogs, a different search area was used for subsequent dogs. The hides were in place for a minimum of 15 min and a maximum of 30 min before the team searched the area.

**FE procedures** Each FE began with the Evaluator orienting the handler, without the dog present, to the area, including identifying and defining boundaries from outside of the search area and reviewing the protocol with the team, including range of number of targets, minimum and maximum height of targets, and time limit. The handler was instructed that when they called an alert, they had to define the location of the odor within a 25 cm radius around the hide’s actual location to be correct. Handlers were given free choice on how they did this, for example, verbally declaring “alert” and pointing at the location. The handler was given 5 min to view the area, assess the conditions, and ask clarifying questions. They were not permitted to enter the designated search area during this time. When ready, the handler returned to the FE area with their dog on a leash; the handler could approach any search area boundary, work in any direction, and work on or off the leash. The handlers could carry the dog’s reinforcer with them and were permitted to provide it within the search area, however, and whenever they deemed appropriate.

**FE test.** The FE was a one-trial event with a total time of 300 s, although there was no penalty for the handler calling an end to the search at any time. The Evaluator began the timer when the dog or the handler stepped inside the search area. The handler was responsible for determining if the dog showed an alert. When a handler called an “alert” and identified the area, the Evaluator verbally confirmed whether the location was correct with either a “yes” or a “no”. An incorrect location was counted as a false alert. At the end of 300 s, the Evaluator called “time”, and the search ended. Any alerts called after the time were not counted, even if the dog had been at the location prior to the period ending. After the search ended, the Evaluator reviewed the score sheet with the handler. The SLF training aids and MB were removed, and the area was left unused for a minimum of 30 min before setting up a new, different FE. No other cleaning or changes were made except to remove identified safety hazards.

**Measurement.** The Evaluator recorded the wind speed and temperature before each FE trial. The time elapsed from the beginning of the search to the handler’s alert call at each location was also recorded. Any alerts called at locations where no SLF training aids were placed were inspected for the presence of SLF egg masses. If SLF egg masses were found, the alert was reclassified from false to correct. To pass the FE, the team was required to locate the total number of hides minus one (*n* − 1), where (*n*) = 3, 4, or 5 and could not have more than one false alert at a location without SLF-training aid. Teams failing to meet these criteria were allowed two additional opportunities to retake the test, either on the same date or at a future date. A different search area was used for retests to prevent familiarity with the prior setup.

#### Analysis

For the ORT and FE, we recorded the dogs’ responses to each trial as either a true positive (TP), false negative (FN), correct rejection (CR), or false positive (FP). Table 1 illustrates a standard confusion matrix, showing how responses are classified in relation to the target’s presence. Trials containing SLF egg masses are referred to as “hot” trials, while trials with no SLF egg masses are classified as “blank” trials.

**Table 1:** Standard confusion matrix for classification of responses.

	**Target present**	**Target absent**
**Target detected**	True positive (TP)	False positive (FP)
**Target not detected**	False negative (FN)	Correct rejection (CR)

Incorrect responses were either false negatives or false positives. A False Negative (Hot Trial Error): The team failed to correctly identify the target during a hot trial by calling an all-clear or selecting an incorrect odor. A False Positive (Blank Trial Error): The team incorrectly identified a non-target odor as the target during a blank trial (Wickens, 2002) We use the following formulas to calculate sensitivity, specificity (ORT only), and precision in the results. Specificity can only be determined when the distracting odors are controlled, as in the ORT array. In the FE, distracting odors include all environmental elements except the target.

\begin{eqnarray*}\text{Sensitivity}= \frac{\text{TP}}{\text{TP}+\text{FN}}  \text{Specificity}= \frac{\text{CR}}{\text{CR}+\text{FP}}  \text{Precision}= \frac{\text{TP}}{\text{TP}+\text{FP}} \end{eqnarray*}

\begin{eqnarray*}\text{Accuracy}= \frac{\text{TP}+\text{CR}}{\text{Total Trials}} \end{eqnarray*}

### Results

#### Participants

The initial call for participants generated 1,033 responses *via* a Google form, with 427 (41.3%) respondents self-identifying as having prior odor detection training with their dog. From these, 110 teams were selected and organized into 12 groups based on geography, proximity, and existing working relationships to form Cohort 1. An additional 72 teams were selected and organized into six groups using the same metrics to form Cohort 2. In total, 182 teams were enrolled, comprising 150 handlers and 182 dogs. Among the handlers, 121 enrolled with one dog, 27 enrolled with two dogs, one with three dogs, and one with four dogs. Of the 182 enrolled dogs, 76 were male, 60 were female, and 46 did not report sex. For dogs with known or reported ages, the youngest was 1 year old, the oldest was 11 years, and the mean age was 5.7 years. Table 2 shows the distribution of breeds. All dogs that attempted at least one ORT had their breed, age, and sex reported.

**Table 2:** Breed distribution of enrolled dogs.

**Breed**	**Number**	**Breed**	**Number**
Australian Koolie	3	Havanese	1
Australian Shepherd	9	Hound Mix	2
Beagle	1	Husky Mix	2
Beauceron	2	Jack Russell Terrier	1
Belgian Malinois	1	Labrador Retriever	11
Bloodhound	1	Lancashire Heeler	1
Border Collie	8	Mixed Breed	21
Boston Terrier	1	Miniature Poodle	1
Brittany	2	Nova Scotia Duck Tolling Retriever	1
Cardigan Welsh Corgi	2	Otterhound	1
Cavalier King Charles Spaniel	1	Parson Russell Terrier	1
Chow Chow	1	Pembroke Welsh Corgi	3
Cocker Spaniel	2	Petit Basset Griffon Vendeen	1
Smooth Collie	1	Pit Bull	2
Dachshund	1	Poodle	4
Dachshund Miniature	1	Portuguese Water Dog	1
Dalmatian	2	Rough Collie	1
Doberman Pinscher	4	Shetland Sheepdog	6
English Shepherd	2	Soft Coated Wheaten Terrier	1
English Springer Spaniel	2	Staffordshire Bull Terrier	2
Flat Coated Retriever	1	Tibetan Terrier	5
German Shepherd	9	Vizsla	1
German Shorthaired Pointer	1	Not Reported	46
Golden Retriever	8		

#### Overall ORT and FE results

**Figure 5:**
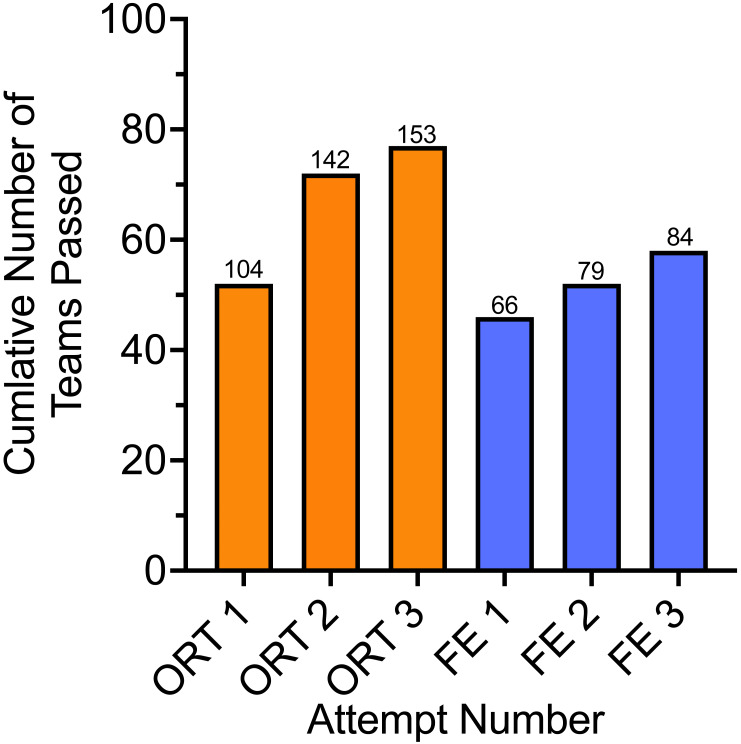
Cumulative number of teams passing the ORT and FE. The *x*-axis represents the attempt number for the ORT and FE. The *y*-axis represents the number of teams with a passing score (80% or greater). The numbers above each bar indicate the cumulative total number of LE-ORT attempts made. Teams had to complete the ORT before progressing to the FE.

Of the 182 teams enrolled, 78 (42.9%) dropped out before making their first attempt at the ORT. Of the remaining 104 teams (57.1%) that attempted the ORT, 77 (42.3% of the original 182 enrolled, 74.0% of the 104 that attempted at least one ORT) successfully passed the ORT. Each team had three attempts to complete the ORT; 52 (50.0%) teams completed the ORT on attempt one, 20 (19.2%) on attempt two, and five (4.8%) on attempt three for a total of 153 ORT’s. Teams that dropped before or between ORT attempts most frequently cited time commitments and scheduling, although not all gave a reason. During 23 ORT tests, the team did not complete all 10 trials. The decision not to complete all 10 trials was at the handler’s discretion, and the reason was not recorded. These attempts, recorded as “Did Not Complete”, were counted as failed attempts at the ORT. All attempts, whether successful or unsuccessful, are included in the following analysis (Fig. 5). The Did Not Complete trials were omitted from analysis beyond Fig. 5, and all further analyses involve the 130 completed ORTs.

Of the 77 teams that passed the ORT, 66 (85.7%) teams attempted the FE, and 56 (72.7%) successfully passed the FE. Each team had three attempts to complete the FE. Forty-six teams completed the FE on attempt one, six on attempt two, and four on attempt three, for a total of 84 FE trials.

#### ORT and FE sensitivity, specificity, and precision

For the 104 teams (total of 130 ORT trials) that completed at least one ORT, we calculated the sensitivity, specificity, and precision (Table 3). For the 66 teams (a total of 84 FE trials) that completed at least one FE, we calculated the sensitivity and precision (Table 3). Calculations used are provided in the analysis section.

**Table 3:** Sensitivity, specificity, and precision. The table shows calculated values for sensitivity, specificity, and precision. Specificity cannot be calculated in the FE as the number of distracting odors in the natural environment is unknown; therefore, the correct rejection rate is impossible to determine.

**Evaluation**	**Sensitivity**	**Specificity**	**Precision**
ORT	0.82	0.57	0.87
FE	0.61	N/A	0.91

#### ORT version and trial effects

**Figure 6:**
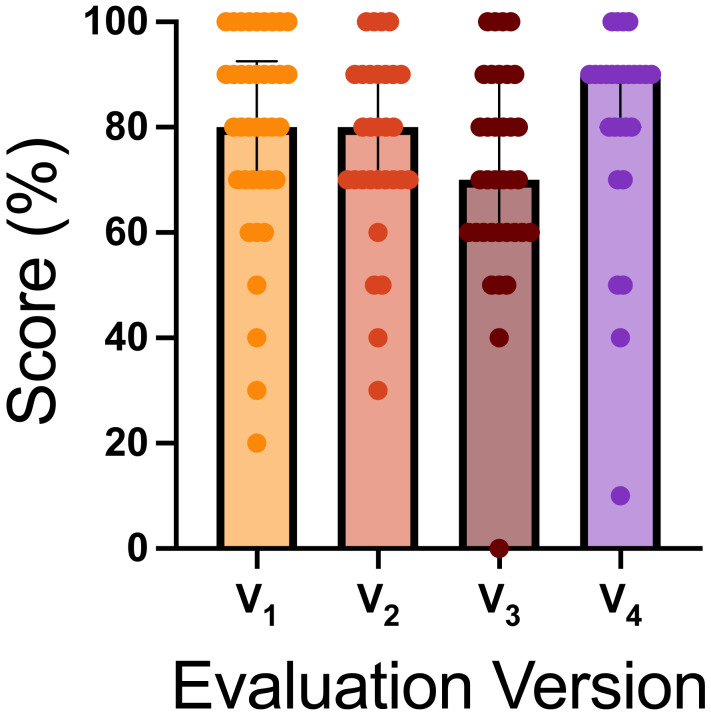
ORT evaluation version scores. The graph shows the score distribution across the four ORT versions, which differed in the number of blank trials: versions one and two had two blank trials, version three had three blank trials, and version four had one blank trial. The bar represents the mean score for that version, the bars represent the standard deviation, and the dots show the individual scores on each version.

The ORT included four versions of odor placement randomization, each containing between one and three blank trials. We investigated whether dogs’ performance varied across versions. Figure 6 displays the scores for each ORT version. Versions one and two included two blank trials, version three had three blank trials, and version four had one blank trial. Using a Kruskal–Wallis test, we found no significant differences in scores, H(3) = 7.226, *p* = .0650, between test versions (Fig. 6).

**Figure 7:**
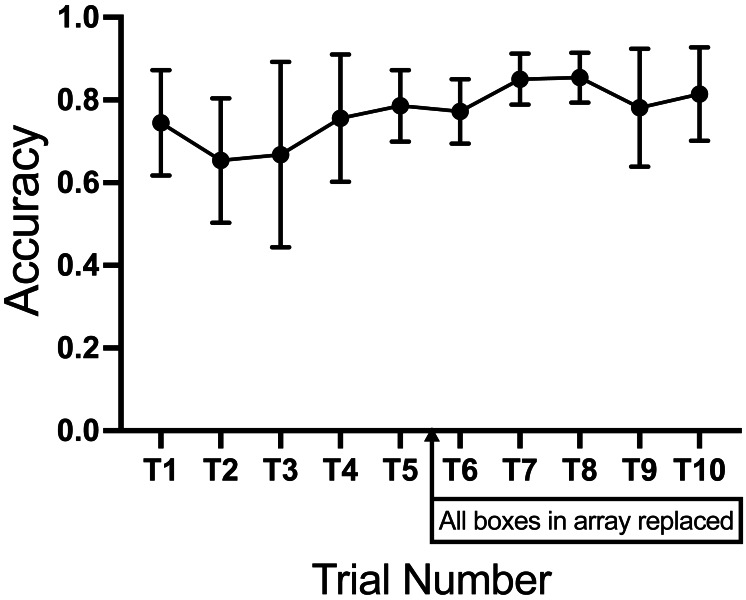
Proportion of correct responses (accuracy) per trial. The graph shows the mean and standard deviation of all versions for correct responses (true positives and correct rejections). Calculation provided in ‘Analysis’.

Of the 1,300 individual trials (130 ORTs with 10 trials each), 901 (76%) trials were correct identifications, either as a true positive (the dog selected the box containing SLF-training aid during a hot trial) or as a correct rejection (the dog made no selection during a blank trial). Of the 281 incorrect responses (23%), 172 (61%) were false negatives (failure to select the correct box in a hot trial), and 102 (36%) were false positives (selection of an incorrect box during a blank trial). Five trials (0.4%) resulted in no response (timed out). Using a Kruskal–Wallis across trial numbers, we found no significant difference in the proportion of correct responses, H(10)=10.15 *p* = .0.3382, between trial numbers (Fig. 7).

#### ORT incorrect responses

**Figure 8:**
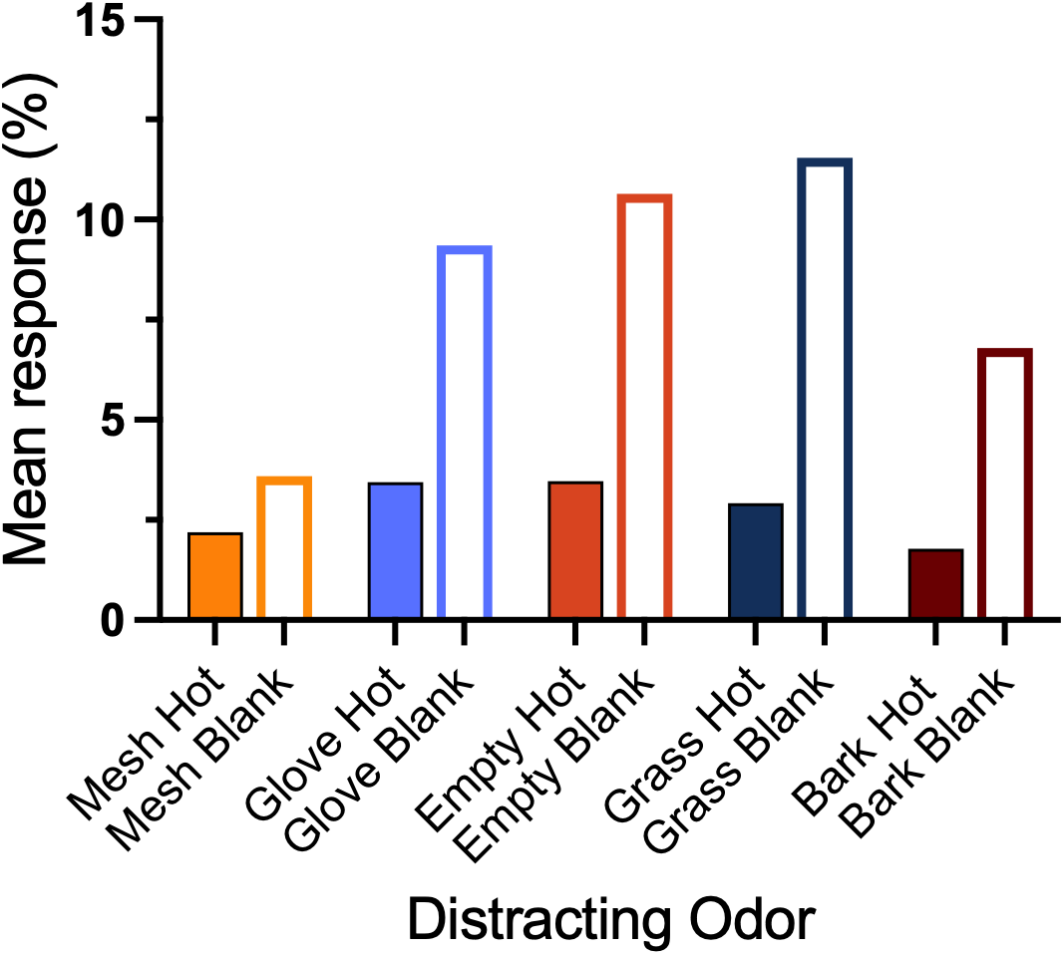
ORT allocation of incorrect responses. The graph shows the mean percent allocation of responses across dogs to the distracting odors in both hot and blank trials.

Of the 1,300 individual trials, 1,032 (79.38%) were hot trials (the SLF target odor was present). Of these 1,032 hot trials, teams gave 186 (18%) false negative responses. Of those trials with a false negative outcome, 55 (29.6%) were called “all-clear” by the handler. Included in the false negative responses are four trials in which the dogs made no response to any odor within the 90-second trial time (*i.e.,* they timed out). The remaining 131 responses were to one of the distracting odors (Fig. 8). The remaining 268 (20.6%) individual trials were blank trials. Of these 268 blank trials, teams gave 118 (44.02%) false positive responses to one of the distracting odors. Included in the false positive responses is one trial where the team timed out (gave no response) (Fig. 8). In each ORT trial, there were either five or six distracting odors. The distribution of the distracting odors varied by the version number. We calculated each team’s allocation of responses to each distracting odor in 10 trials, separated by hot trials *versus* blank trials as:

\begin{eqnarray*} \frac{\text{Number of Responses to Odor X}}{\text{Total Number of Presentations of Odor X}} \times 100\%. \end{eqnarray*}

#### ORT time searching

**Figure 9:**
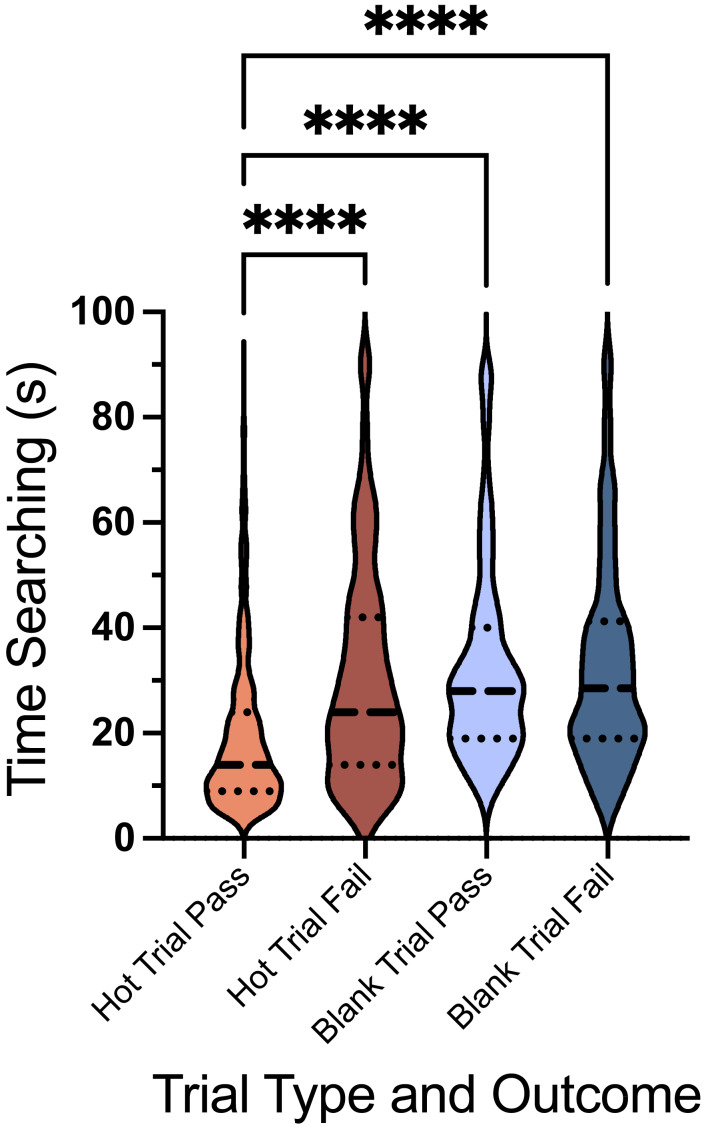
Time searching for hot trials and blank trials. The violin plot displays the median (dashed line), along with the 25th and 75th percentiles (dotted lines), showing the time in seconds spent searching during hot trials and blank trials, separated by pass and fail outcomes. The width of the plot at each point reflects the distribution density of the data at that *Y*-axis value (Time (s)) **** *p* < 0.0001.

We recorded each team’s time spent searching the array, from when they crossed the start line to when the handler notified the Evaluator of their determination. Any team that timed out was recorded as a fail, with a search time of 90 s (the maximum allowable time per trial). For analysis, we separated the trials into four categories: hot trial correct response (Hot Trial Pass), hot trial incorrect response (Hot Trial Fail), blank trial correct response (Blank Trial Pass), and blank trial incorrect response (Blank Trial Fail). The median values (s) and inter-quartile range were as follows: Hot Trial Pass (*Med* = 14, *IQR* = 87), Hot Trial Fail (Med = 24, IQR = 88), Blank Trial Pass (*Med* = 28, *IQR* = 82), and Blank Trial Fail (*Med* = 28.5, *IQR* = 86). A Kruskal–Wallis test revealed a significant effect of trial type, H(4) = 17831, *p* < 0.0001. Dunn’s *post hoc* test indicated that the time spent searching was significantly greater during failed hot trials compared to passed hot trials (*p* < 0.0001), significantly greater during passed blank trials compared to passed hot trials (*p* < 0.0001), and failed blank trials compared to passed hot trials (*p* < 0.0001) (Fig. 9).

#### FE effects of wind and temperature

For each FE trial, we recorded the temperature (°C) and wind speed (km/h). We used a multiple logistic regression to evaluate wind speed and temperature as predictors of the likelihood of passing the FE. The dependent variable was the outcome of the FE (0 = fail, 1 = pass). The model included wind speed and temperature as independent variables. The model did not significantly predict FE outcomes, as neither wind speed (*β* = 0.021, SE = 0.035, 95% CI [−0.046–0.095]) nor temperature (*β* = 0.011, SE = 0.030, 95% CI [−0.048–0.071]) were statistically significant. The odds ratios for wind (*OR* = 1.021, 95% CI [0.955–1.099]) and temperature (*OR* = 1.011, 95% CI [0.954–1.074]) indicate minimal influence of these variables on passing outcomes. Model diagnostics further suggest poor predictive performance. The area under the receiver operating characteristic (ROC) curve was 0.590 (*SE* = 0.071, 95% CI [0.451–0.730], p = .179), indicating that the model performed only slightly better than chance.

## Experiment 2—transition to live SLF eggs

Teams that passed the FE could participate in Experiment 2—Transition to Live SLF Eggs. This experiment aimed to evaluate whether a participatory science dog team, trained on a devitalized odor, could transition to a live version of the same odor and maintain its sensitivity and precision.

### Methods and materials

The Live Egg Odor Recognition Test (LE-ORT) followed the same protocols as the ORT in Experiment 1 (refer to ‘Odor Recognition Trial’, with four key modifications:

• During the two-minute acclimation period, the dogs were first exposed to the live SLF egg odor. As in the ORT, the handler knew which of the three acclimation boxes contained the target odor.
• The number of trials was reduced from 10 to five.
• No trials were blank; all trials included live SLF egg samples.
• The box arrays included only live eggs, with no devitalized samples.

#### Analysis

For the LE-ORT, all trials included the target; we recorded the dogs’ responses to each trial as either a true positive (TP) or a false negative (FN). Table 1 illustrates a standard confusion matrix, showing how responses are classified in relation to the target’s presence. A passing score required 80% correct responses. The calculations used are provided in Experiment 1, analysis section.

### Results

#### Overall LE ORT

**Figure 10:**
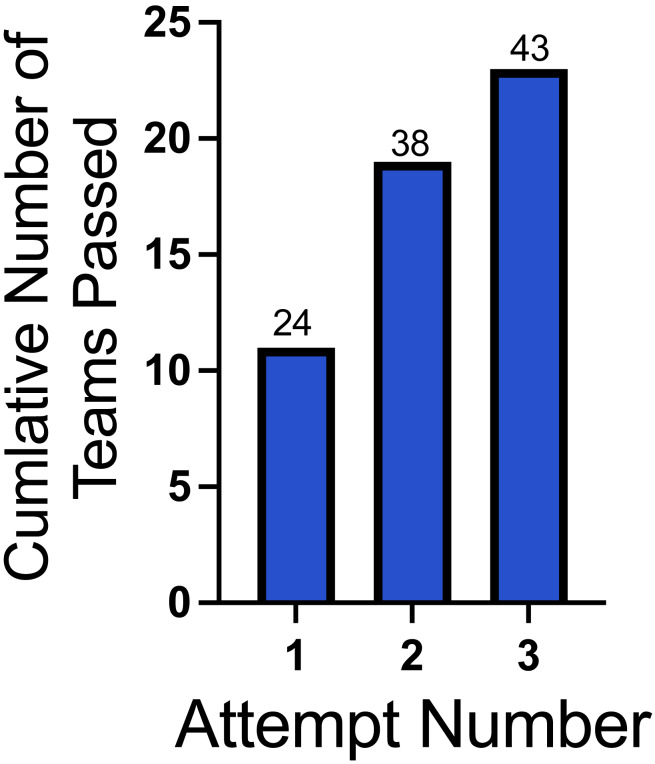
Cumulative number of teams passing the LE-ORT. The graph shows the progression of teams meeting the passing criteria across successive attempts for the ORT evaluation. The *x*-axis represents the attempt number, while the *y*-axis displays the cumulative number of teams passing each test. The numbers above each bar indicate the cumulative total number of LE-ORT attempts made.

Of the 56 teams that completed the ORT and FE in Experiment 1, 24 were available when live SLF eggs were accessible. Of the 24 teams that attempted the LE-ORT, 22 teams (91.7%) passed. Each team had three attempts to complete the LE-ORT. 11 teams passed the LE-ORT on attempt one, eight on attempt two, and four on attempt three, for a total of 29 LE-ORTs. Figure 10 shows the cumulative number of teams passing. During 14 LE-ORT attempts, the team did not complete all five trials. These attempts, recorded as “Did Not Complete”, were counted as a failed attempt at the ORT but were excluded from subsequent analysis. All attempts, whether successful or unsuccessful, are included in the following analysis.

#### LE-ORT sensitivity

We calculated the sensitivity of the 24 teams that completed at least one LE-ORT. The sensitivity for the LE-ORT was 0.8.

#### LE-ORT time searching

**Figure 11:**
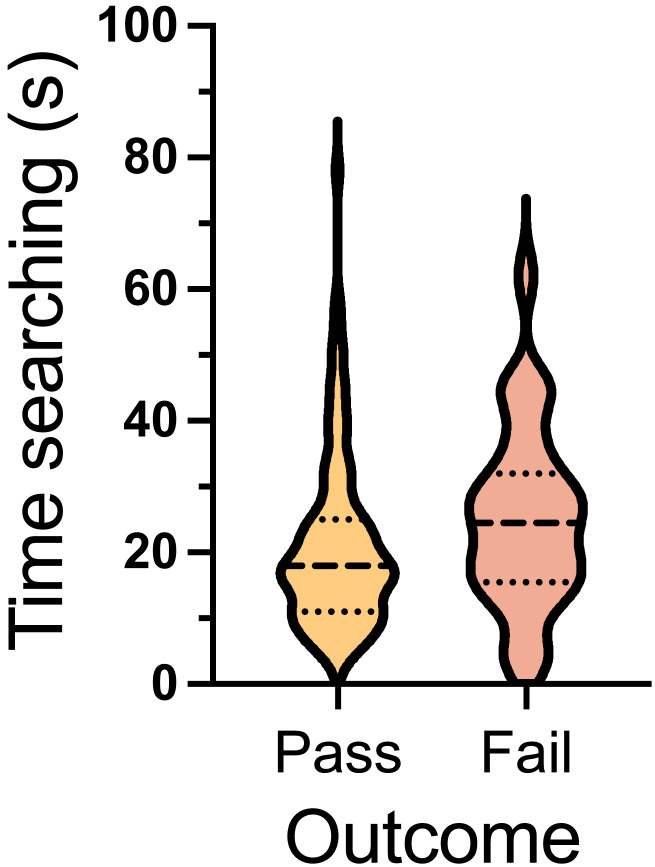
Time searching for LE-ORT pass and fail trials. The violin plot displays the median (dashed line) along with the 25th and 75th percentiles (dotted lines), showing the time in seconds spent searching during hot trials and blank trials, separated by pass and fail outcomes. The width of the plot at each point reflects the density of the data distribution at that *Y*-axis value (time in seconds).

A Mann–Whitney U test was conducted to compare the time spent searching between passing and failing teams in hot trials. The results indicated no significant difference in searching time between passing and failing trials. *U* = 1268, *p* = 0.0632 (Fig. 11).

## Discussion

We investigated whether participatory science dog teams, trained using devitalized SLF egg masses, could reliably detect these targets and generalize their skills to live SLF egg masses. Our results demonstrate the feasibility of these teams in successfully detecting SLF egg masses using devitalized training samples and generalizing them to live samples. These findings highlight the potential of participatory science teams to complement professional CDD programs, offering scalable and impactful solutions for managing invasive species. The high level of interest from handlers (1,033 handlers expressed an interest) in participating in this study suggests that community scientists, particularly those in the sport detection dog community, are eager to contribute to projects with meaningful environmental outcomes. This enthusiasm supports the scalability of participatory science programs and underscores the untapped potential of sport detection dog teams as valuable resources for addressing critical conservation challenges. For this study, we selected geographically proximate handlers with prior relationships established within training groups. The total number of enrolled teams was limited to 182 due to constraints in accessing and distributing SLF egg masses as training aids. As is common in participatory science projects, a participant attrition rate of 47% was observed. This attrition could partly be attributed to the unanticipated time commitment required for training and evaluation. While we attempted to clearly communicate the potential time investment during recruitment, we also needed to balance this with effective enrollment strategies. Other possible reasons for attrition include unforeseen changes, such as a dog becoming ill or passing away, the dog’s lack of interest in training due to a new odor, or personal life circumstances. Unfortunately, understanding the exact reasons for attrition is challenging, as participants who have stopped responding to the study are often reluctant to complete exit surveys. Finally, participants were recruited exclusively through social media. It is possible that targeted recruitment efforts focused on handlers with a demonstrated interest in training their dogs on conservation odors could result in higher retention rates.

The results from both experiments highlight that with prior experience in odor detection, participatory science dog teams of various breeds, can effectively learn to detect novel, agriculturally relevant target odor from devitalized SLF egg masses. These findings are consistent with prior research (Essler et al., 2021; Aviles-Rosa et al., 2023; Williams & Johnston, 2002; Waggoner et al., 2022), which demonstrated that dogs could successfully transition to detecting new odors with appropriate training. Notably, this study’s training process was entirely handler-driven, with no standardized training protocol or direct influence from researchers. This approach demonstrates the feasibility of community-led training and highlights the potential for participatory science teams to make meaningful contributions to real-world conservation efforts. In Experiment 1, of the 95 teams that completed at least one attempt at the ORT, 73.7% passed, and 60% (of the 95) subsequently passed the FE. Scheduling the ORT and FE emerged as the greatest challenge to ensuring continued participation and retesting. Both evaluations required significant preparation, including securing an appropriate space, arranging helpers to manage cameras, and setting up odor boxes. Whether the researchers or their designees conducted the assessments, many teams cited the lack of scheduling flexibility as a primary reason for not initially testing or retesting after a failed attempt. Addressing these logistical barriers in future studies could lead to higher participation rates and improved overall pass rates. We selected the ORT as the primary evaluation method because it aligns with professional detection dog practices (Dogs and Sensors Consensus Body, 2021), assessing the dog’s ability to process olfactory stimuli, discriminate between distractor odors and the target, and indicate either the presence of the target or an all-clear. As a highly controlled evaluation of the dog’s understanding of the trained odor profile, the ORT provides a quantifiable outcome that may serve as a good indicator of field performance. However, this study did not compare the field performance of dogs that passed the ORT to those that did not, as this was beyond the scope of our focus. Therefore, while we cannot conclude the ORT’s predictability for field performance, the results do allow us to evaluate the dogs’ ability to be trained to detect a specific odor.

The controlled environment of the ORT also facilitated the calculation of sensitivity, specificity, and precision, offering a robust screening tool for potential field applications. We found that the teams’ detection sensitivity for the ORT was 0.82, indicating that teams could reliably detect devitalized SLF egg mass odors within a six-sample array if the target were present. Sensitivity was calculated as the proportion of true positives (TP) out of the total number of actual positive trials (TP + FN). To advance in the study, a team had to achieve a passing score, which was set at 80%. This means that teams needed to correctly identify the target or reject a blank trial in at least 80% of trials to meet the performance threshold.

Challenges related to the trial duration (90 s per trial with a 120-second inter-trial interval for 10 consecutive trials) and the repetitive nature of the task may have influenced performance, suggesting that additional task-specific training could further enhance sensitivity. For example, the ORT array can be used as a training component. In contrast, sensitivity dropped to 0.61 in the FE, a decline consistent with the added challenges of a novel outdoor environment, limited search time (5 min per trial), and the subtle odor of small SLF samples (0.5 g). Despite this, the FE sensitivity aligns with prior studies, which report acceptable levels as low as 25% when compared to alternative detection methods (Mathews et al., 2013; Moser et al., 2020). Specificity, assessed only in the ORT, was 0.57, reflecting the difficulty of correctly identifying blank trials in a controlled testing environment. Among the four ORT versions, those with more blank trials tended to have lower average scores (Fig. 6), though statistical differences were not observed. Additionally, teams required more time to complete blank trials compared to hot trials (Fig. 9), potentially reflecting the increased demand of ruling out distractor odors, the influence of the handler, or a lack of familiarity with blank trials, as well as the time pressure of each trial.

Finally, precision which evaluates the proportion of positive identifications that are correct (*i.e.,* the accuracy of true positive responses) was 0.87 in the odor recognition test (ORT) and increased to 0.91 in the field evaluation (FE). Because participation in the FE required successful completion of the ORT, the teams evaluated in the FE represented a more proficient subset of the original sample. The lower precision observed in the ORT likely reflects false-positive responses during blank trials, in which no SLF target odor was present. These blank trials made it more challenging for dogs and handlers to confidently rule out distractor odors. In contrast, the FE did not include blank trials the SLF target odor was always present resulting in a more predictable search scenario. This likely contributed to the higher precision observed in the FE, as the absence of blank trials reduced the probability of false-positive responses. For real-world applications, where participatory dog teams might screen shipped goods or orchards for invasive species, even detecting a single egg mass could be highly impactful. A detection would prompt further inspection by human searchers or professional detection dog teams under more controlled conditions. The high precision observed in the FE demonstrates the potential reliability of participatory dog teams in these scenarios.

The reported sensitivity and precision indicate that participatory science dog teams can effectively identify SLF egg masses in realistic environments despite the competing environmental odors. This capability is critical given the cryptic nature of SLF egg masses, which are often located on concealed surfaces or in inaccessible areas.

During the FE, teams worked in a more naturalistic setting and were subject to the weather conditions on the test day. We recorded wind speed and temperature at the beginning of each FE and found that neither variable predicted a pass. This finding suggests that, within the range of environmental conditions encountered during this study, teams can search effectively, and dogs can detect the odor. However, it is possible that extreme temperatures or wind conditions, which were not observed during this study, could influence performance. Additionally, individual differences among dogs may play a role, with dogs trained and habituated to high or low temperatures potentially performing better in those conditions than dogs with less exposure to such temperatures. Similarly, dogs accustomed to training in high or variable wind conditions may have an advantage over those with limited experience in such environments.

All ORT, FE, and LE-ORT trials were masked to the handler and any observers to eliminate potential bias from the handler. Handler influence is a critical factor in detection dog teams, as it can impact both the dog’s performance and the interpretation of results. Research in professional detection dog settings has demonstrated that, despite rigorous training to minimize bias, handler influence can still occur (Clark, Sibbald & Rooney, 2020). Many participants in this study came from backgrounds in sports detection or volunteer search and likely lacked formal training in mitigating handler influence. However, these fields often include testing scenarios where the outcome is unknown to the handler, providing some experience with masked searches. This highlights the need for targeted education on reducing handler bias and increased practice in fully masked search scenarios, ensuring that detection outcomes are driven solely by the dog’s capabilities.

The challenges posed by blank trials in the ORT highlight the complexities of handler influence and the importance of targeted training. Teams required significantly more time to complete blank trials than hot trials, suggesting that ruling out distractor odors when no target was present was more cognitively demanding for both the dog and handler. This could also reflect variability in the frequency or emphasis placed on blank trials during training. Preliminary research by Ken Ramirez suggests that incorporating an “all-clear” response into detection dog training protocols may improve specificity and reduce handler uncertainty during blank trials (Ramirez, 2023). However, this approach has not been widely studied yet. In this study, we did not require teams to train or utilize a specific all-clear response, which may have contributed to the observed variability in performance. Instead, handlers were responsible for interpreting their dog’s behavior and determining whether the array was blank or the dog had failed to locate the target. This reliance on handler interpretation highlights the need for further research into the impact of formal all-clear training and the development of strategies to enhance handler decision-making in ambiguous scenarios, such as blank trials.

Despite these challenges, the results of this study remain robust. The dogs’ abilities were demonstrated across the ORT and FE, as evidenced by their sensitivity, precision, and overall performance. Importantly, this study did not require a specific trained response to the target odor or ask handlers to describe the behavior they interpreted as indicative of a find. Without a discrete, trained response, some handlers likely misinterpreted their dogs’ behavior, which may have led to missed opportunities to identify the target correctly. This suggests that teams might have achieved even higher success rates with a more accurate interpretation of their dogs’ signals. Future work should examine whether the implementation of a required and defined response would alter performance in the ORT or FE.

Conversely, it is highly improbable that handlers guessed correct responses in a way that artificially inflated the dogs’ performance. Several measures were taken to eliminate such possibilities. The boxes used in the ORT were monitored for discernible marks, their placement in the array was randomized, and the boxes were swapped after trial five to eliminate visual cues to the handler and olfactory cues for the dog. These controls ensure that the recorded results primarily reflect the dogs’ abilities, with handler influence playing only a secondary role. Further analysis of video recordings from the ORT and FE could provide valuable insights into discrepancies between the dogs’ behavior and the handlers’ interpretations, particularly during blank trials. This could inform strategies to enhance handler training and improve communication between handlers and their dogs. For example, incorporating specific training on blank trials and teaching an all-clear response could further improve performance and reduce variability in participatory science detection teams.

We found that dogs trained on devitalized samples could transition to the odor of SLF eggs from devitalized to live samples, a critical step with significant implications for training CDDs for SLF egg mass detection. Training with devitalized samples minimizes the risk of inadvertently introducing live SLF eggs into new areas during the training process, ensuring safer and more responsible operational practices. The 24 teams that participated in the LE-ORT had no prior exposure to the live SLF egg odor before the 2-minute acclimation period and the LE-ORT. Of these, 92% successfully passed, demonstrating the dogs’ ability to rapidly learn the target odor from devitalized to live SLF eggs. The calculated sensitivity for the LE-ORT was 0.8, consistent with the sensitivity observed in the ORT trials. This finding supports the results of Essler et al. (2021), which showed that while dogs did not spontaneously generalize to live SLF eggs, their performance improved with repeated exposure. With a brief additional training period on live SLF eggs, more teams would likely pass the LE-ORT and potentially increase sensitivity. These findings further highlight the practicality and safety of using devitalized SLF egg masses as an effective training tool for detection dog teams.

In the LE-ORT, all trials were hot, and no significant difference in search times was observed between passing and failing trials. This consistency in search times may reflect improved stability in the dogs’ performance during this test. Further investigation is needed to identify additional factors contributing to this stability. Possible explanations include reduced handler influence, the absence of blank trials, and the smaller number of total trials compared to the ORT. Considering the consistent search times and the high sensitivity observed, this test appears to have primarily assessed the dogs’ ability to recognize and respond to the target odor rather than their ability to discriminate between target and non-target odors or manage uncertainty in blank trials.

## Conclusions

Several key findings emerged across the experiments. First, the evaluation criteria developed in this study provide a robust and quantifiable metric for assessing participatory science dog detection programs, helping to bridge the gap between community science and professional detection teams. The current literature lacks consensus on standardized metrics for evaluating CDDs in fieldwork (McKeague, Finlay & Rooney, 2024). Sensitivity, specificity, and precision are commonly reported, but the contexts in which these measures are achieved are frequently unclear (McKeague, Finlay & Rooney, 2024). Furthermore, no national proficiency guidelines exist for professional CDD teams before field deployment. Sensitivity, specificity, and precision are recommended for controlled environments, whereas sensitivity, accuracy, and efficiency are prioritized for naturalistic settings (Bennett, Hauser & Moore, 2020). We hope this work will serve as a basis for evaluating the effectiveness of CDD teams and those studying their operations.

Second, blank trials in the ORT posed notable challenges, as indicated by increased search times and lower specificity compared to hot trials. These findings highlight the increased demands on dogs to distinguish between target odors and distractor odors, as well as the potential influence of handlers in ambiguous scenarios. However, prior research and practical experience suggest that training dogs with masked trials can quickly improve their ability to confidently reject non-target odors, thereby reducing search times and increasing specificity. Third, using devitalized SLF egg masses as training tools represents a practical, safe, and scalable approach for expanding detection efforts without the risk of spreading invasive species

Collectively, our findings support previous research demonstrating that trained dogs outperform humans in locating SLF egg masses (Keller & Hoover, 2023), and underscore the potential for participatory science dog teams to serve as a scalable supplement to professional detection efforts in conservation contexts. While the sensitivity of participatory teams in the FE was lower than in the controlled environment of the ORT, it remained sufficient to identify SLF egg masses at a rate that outperforms alternative detection methods. These results suggest that, with targeted training and support, community scientists and their dogs can play an invaluable role in managing invasive species. Future research should address challenges posed by blank trials, develop standardized neutral response protocols to minimize false positives, and implement strategies to reduce handler interpretation bias such as structured feedback or decision-point training. Additionally, enhancing teams capacity to operate across varied environments remains a key priority for broader conservation and agricultural protection efforts. Notably, this study experienced a relatively high dropout rate, which may reflect the variability in individual motivation, access to local training support, or logistical constraints. Future studies should explore mechanisms to improve participant retention, such as offering remote mentorship, establishing clearer performance benchmarks, or creating community-based support networks. Lastly, further work is needed to establish a reliable, scalable process for connecting trained participatory science teams with stakeholders in need of detection services.

This study provides proof of concept for integrating participatory science dog teams into conservation detection programs. By leveraging devitalized SLF egg masses as training tools, we demonstrated that community-based teams can be effectively prepared without risking the spread of invasive species. The high public interest in this effort underscores the potential for community science programs to engage communities in meaningful environmental conservation initiatives. These findings lay the groundwork for broader collaboration between community scientists and stakeholders, supporting future efforts to protect ecosystems and agricultural systems from invasive threats.

## Additional Information and Declarations

### Field Study Permissions

The following information was supplied relating to field study approvals (i.e., approving body and any reference numbers): Virginia Tech IACUC. Approval number: 23-025.

### Data Availability

The following information was supplied regarding data availability: All data are available at the Virginia Tech Library Data Repository: Dickinson, Sally; Feuerbacher, Erica; Nita, Mizuho; Hall, Nathaniel; Aviles-Rosa, Edgar O. (2025). Spotted Lanternfly detection data - ORT, FE and LEORT. University Libraries, Virginia Tech. Dataset. https://doi.org/10.7294/28390157.v1.
